# Where did bone come from? An overview of its evolution

**DOI:** 10.3109/17453674.2011.588861

**Published:** 2011-09-02

**Authors:** Darja Obradovic Wagner, Per Aspenberg

**Affiliations:** ^1^Institute of Chemistry and Biochemistry, Freie Universität Berlin, Berlin, Germany; ^2^Orthopedics, AIR/IKE, Faculty of Health Science, Linköping University, Linköping, Sweden; Correspondence: darja_obradovic@yahoo.de

## Abstract

Bone is specific to vertebrates, and originated as mineralization around the basal membrane of the throat or skin, giving rise to tooth-like structures and protective shields in animals with a soft cartilage-like endoskeleton. A combination of fossil anatomy and genetic information from modern species has improved our understanding of the evolution of bone. Thus, even in man, there are still similarities in the molecular regulation of skin appendages and bone. This article gives a brief overview of the major milestones in skeletal evolution. Some molecular machineries involving members of core genetic networks and their interactions are described in the context of both old theories and modern genetic approaches.

## Skeletal evolution: different views

If this article had been written a decade ago, it would have been considerably different. Given that most primitive examples of mineralization belong to extinct lineages, for a long time our understanding of bone evolution was entirely based on the available fossil evidence. Only paleontology studies offered the possibility of gaining some insight into the ancient processes that led to mineralized skeleton; from the evidence available, it was surmised that the vertebrates were most likely descended from amphioxus-like forms with a notochord. These were followed by jawless creatures with a cartilage-like endoskeleton, reminiscent of the modern hagfish or lamprey (Holland et al. 2001, Meulemans and Bronner-Fraser 2007). The next big event was the appearance of mineralized skeletal parts; this presented a major evolutionary leap and led directly to the rise of the vertebrate lineage (Denison 1963, Sansom et al. 1992).

Given that primitive fossilized vertebral skeletons are scarce and that their remains often contain tissues that are difficult to classify, the emergence of the four skeletal tissue types (enamel, bone, dentine, cartilage) was controversial. While one hypothesis suggested that the four tissues all emerged early in vertebrate evolution, the other assumed a long time of tissue plasticity in early mineralized skeletons which preceded differentiation processes that came later on (Tarlo 1963, Halstead 1969). In addition, for many decades, synthesis of paleontological data was influenced by Ernst Haeckel's biogenetic law—that ontogeny recapitulates phylogeny—meaning that skeletons of ancestral adult vertebrates were assumed to be derived from cartilage, analogous to their embryonic skeletons (Hall 2003).

Nowadays, evidence of the mineralization of tissues is often related to the repertoire of specific secretory calcium-binding phosphoprotein *(SCPP)* genes present in various vertebrate lineages (Kawasaki and Weiss 2003). Expression analysis revealed *SCPP* genes and combinations of genes that are mainly used in the bone and dentine, while other SCPP variants were found to be used to build up enamel structures. Current studies suggest a close relationship between bone, dentine, and enamel in terms of a mineralized-tissue continuum in which contemporary dental tissues have evolved from an ancestral continuum through lineage-specific modifications (Kawasaki 2009).

Finally, many recent reports view skeletogenesis in light of the evolution of distinct core gene networks that have been essential to vertebrate phylogeny (Erwin and Davidson 2009). For example, a recent search for the molecular origins of skeletal development has attracted attention to the Runt family of genes (*RUNX* 1, -2, and -3). RUNX proteins regulate the key factors involved in skeletogenesis. They are crucial for cartilage development, and RUNX2-deficient mice lack bone (Ito and Miyazone 2003, Fujita et al. 2004). Together with the *RUNX* family, several more newly discovered gene networks are currently seen as central to understanding the evolution of skeleton.

## From outer to inner protection: design combined with fortuitous circumstances

So, how did mineralized tissues develop in the first place? What factors forced the first organisms to develop protective shields?

Following the violent moves of tectonic plates about 1.5 billion (1.5 × 10^9^) years ago, huge amounts of minerals, including CaCO_3_, were washed into the oceans. This created the possibility for its inhabitants of developing hard body parts, such as shells or spines. At first, this helped unicellular organisms to cope with excessive amounts of minerals and to prevent over-crusting. It also led to the sharp increase in the diversity of multicellular organisms (and their fossils!) a little more than 0.5 billion years ago, known as the “Cambrian explosion” (Schopf 1994, Kawasaki et al. 2004). Furthermore, the appearance of a rigid outside skeleton extended the effective length of limbs, thus permitting more rapid locomotion in many organisms. The appearance of mineralized body parts is seen by many scientists as one of the forces that generally increased the pace of animal evolution (Kumar and Hedges 1998, Kutschera and Niklas 2004).

As much as exoskeleton added speed to the evolution of animal life in general and created opportunities for animals to expand their activity radius by using calcified extremities and protection shields, it also imposed limitations, associated mostly with limited body size and lack of surface sensory organs. In addition, rigid shells and shields did not allow much movement and locomotion; therefore, the next major change in the evolution of skeleton—dislocation of mineralized skeleton from the outside to the inside of animal bodies, proved to be a major adaptive advantage. Especially in animal lineages that later gave rise to vertebrates, the appearance of endoskeleton enabled the expansion of activity radius and habitation of entirely new environments (Bennet 1991). In addition, those developments encouraged the development of a strong muscular system and added further adaptive values such as greater overall mobility and the appearance of a regenerative and environment-sensitive outer dermis (Ruben and Battalia 1979, Ruben and Bennett 1980).

## Biomaterial challenges

Another major advantage of the architecture of mineralized skeleton was the development of an attribute of bone that decisively set vertebrates apart from virtually all other multicellular eukaryotes. The hard mineral fraction consisting mainly of calcium carbonate, which had been used over millions of years to build all forms of marine exoskeletons, was replaced by calcium phosphate, mostly in the form of calcium hydroxyapatite (Ruben and Bennett 1981, Ruben and Battalia 1992, Omelon et al. 2009). But why did vertebrates choose an entirely new mineralization strategy, and what special properties of calcium hydroxyapatite led to its integration into early vertebral skeletons? There are several hypotheses for the origin of a phosphate-based skeleton. The first major advantage that may have led early vertebrates to sequester marine phosphate could have been the fact that accessible phosphate stores were useful sources of energy for active animals, and may therefore have improved their metabolism (Ruben and Bennett 1980). However, this view of the unique chemical attributes of vertebrates affords no advantage that would not have been equally advantageous to the invertebrates.

Another possible advantage of the novel chemical composition of vertebrate skeletons might be that calcium hydroxyapatite building blocks provide greater chemical stability. This may have been important, especially in the acidic environments created after bursts or periods of intense physical activity—conditions that are typical of most vertebrate species (Ruben and Battalia 1979, Ruben and Bennett 1981). Following intense activity, vertebrates experience a depression in the pH values of extracellular fluids, dropping in humans from a resting value of 7.41 to a post-activity pH of 7.15. This pathway relies on the production of lactic acid for generation of ATP, and enables vertebrates to attain levels of energy production that would not be possible with aerobic metabolism alone. However, the release of lactic acid and decrease in extracellular pH causes a certain degree of skeletal dissociation and hypercalcemia. As discussed by Ruben and Bennet (1980, 1981), the magnitude of these processes would be significantly greater if the skeleton consisted of calcitic rather than phosphate-based material, which would necessitate a lower overall metabolism and activity. This hypothesis was investigated in a series of in vivo experiments with fish, where calcite or hydroxyapatite crystals were implanted into different body parts and the animals were kept on varying exercise regimes. Subsequent analysis of fish serum and implants showed that at pH 7.1 (associated with high activity), calcium concentration and implant dissolution rates were considerably higher in fish with calcite implants. In summary, other biomaterial properties being equal, hydroxyapatite builds a more stable mineral component of the skeleton than can be achieved with a calcitic material, which is particularly important at pH ranges that are associated with the intense activity and a high-energy consuming lifestyle typical of vertebrates.

## What came first: teeth or shield?

**Figure 1.**
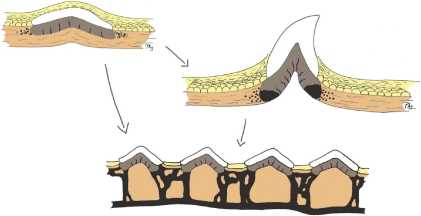
The origin of bone. Precipitation of hydroxyapatite around the basal membrane of the skin gave rise to enamel- and dentine-like tissues that formed odontodes, which became the progenitors of teeth and scales. Spread of mineralization deeper in the dermis formed shields consisting of acellular—and later cellular—bone. (Adapted from Donoghue et al. 2006).

**Figure 2.**
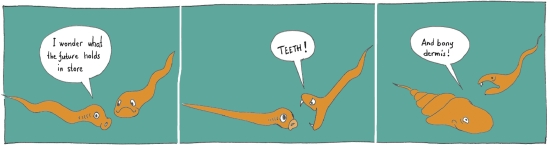
The role of predation.

The earliest mineralized structures in the vertebrate lineage were tooth-like structures, odontodes. It is debated whether these emerged first in the throats of jawless, eel-like creatures with a notochord (conodonts), or as dental-like structures in the skin, arranged closely together to form a protective shield (Figure 1). In either case, it is obvious that predation and protection from predation was a driving force for this development (Figure 2). Modern theories suggest that it is not so meaningful to argue that teeth are the origin of dermal mineralization, or vice versa. Early teeth and the forerunners of bony skin plates appear to be the product of the same genetic machinery, regulating epithelial/mesenchyme interactions and able to produce similar structures at different locations. The machinery, involving BMPs, WNTs, and FGFs, was probably in place for other functions, e.g. forming sensory structures related to modern taste buds, and needed only modifications to enable formation of mineralized structures (Fraser et al. 2010). Thus, in modern animals, the RUNX transcription factors—which are crucial for bone formation—are also involved in the regulation of skin thickness and skin appendages such as hair follicles (Glotzer et al. 2008).

## The next steps in skeletal evolution: from protective shields to endochondral ossification

The earliest skeleton in the vertebrate lineage was a non-collagen-based unmineralized cartilaginous endoskeleton. It was associated mostly with the pharynx, in taxa such as lancelets, lampreys, and hagfish . After the evolution of collagen II from earlier simple collagens, a collagen-based cartilage could form. In contrast to animals with completely non-collagenous skeletons, some of the primitive chondrichthyans (such as sharks) were able to form skeletal parts though the process of endochondral ossification; however, due to the lack of fossil record s, the exact time of origin and the extent to which this mechanism was used is unclear (Hall 2005 and references therein).

From an evolutionary point of view, endochondral ossification is the younger of the 2 types of bone formation (the older dermal bone was formed by intramembranous osssification). It occurs in vertebrate skeletons by replacement of cartilage templates. The process of endochondral ossification evolved gradually, starting with perichondral bone deposition using the molecular tools that had evolved during the evolution of bony shields in the skin. This preceded the evolution of processes of cartilage degradation and endochondral bone deposition, as shown mostly by studies on shark skeletogenesis (Mundlos and Olsen 1997, Eames et al. 2007).

In addition to delivering bone as an organ, endochondral ossification provided a structural support for vertebrate limb development. However, there is still debate and uncertainty concerning the transition from fish fins to vertebrate limbs. Did limbs first develop in aquatic animals, thus predisposing them to walk on land? Did digits appear in the water, or do they represent an adaptation to terrestrial environments? What was the original number of digits? The pair of limbs that came first, and also many details about their embryonic development are awaiting more definite answers (Hell 2005, and references therein). A recent study suggested that it is mostly the loss of the actinodin gene family (this family encodes proteins making up the rigid fibers of fins) which might explain how fish evolved into four-limbed vertebrates (Zhang et al. 2010). These authors' genetic experiments on zebrafish showed that it was probably a loss of only a small number of genes that acted as a creative force in evolution, accounting for the huge evolutionary transition from fins to limbs.

With the advent of terrestrial vertebrates, skeletal function expanded in new directions. Although bone was still a reservoir of calcium and phosphorus, and acted as a shield for vulnerable body parts, it also began to serve as a site of blood cell production, and allowed movement and mechanical support.

## Skeletal evolution: from fossils to gene networks

**Figure 3.**
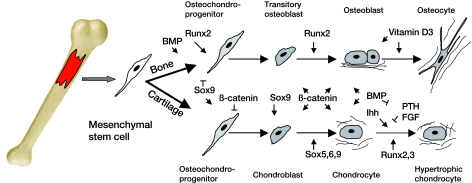
Major gene networks that govern skeletal evolution. Arrows indicate positive interactions and horizontal lines indicate negative interactions. The scheme depicts signaling pathways as they are currently understood, but most of the processes are under intensive investigation. The information has been taken from multiple sources that are cited throughout the text.

While osteoblasts and chondrocytes are derived from mesencymal progenitors, osteoclasts are of hematopoietic origin (Porter and Calvi 2008, Zhang et al. 2009). Cell fate decisions are regulated by a vast array of deeply rooted gene networks, and we are only beginning to understand their hierarchy and interdependence (Figure 3).

One of the major components that enable skeletogenesis is the Runt gene family. For example, *RUNX2* function is central to regulaton of osteoblast differentiation and tooth differentiation, and lack of one of the paired *RUNX2* genes in humans causes a bone disease called cleidocranial dysplasia (Otto et al. 2002). Although our knowledge about the evolutionary origin of this gene network is limited, experimental work has shown that members of the family regulate transcription factors that are essential for skeletogenesis and that are important for cartilage maturation (Hecht et al. 2008). These authors analyzed the presence and the expression of RUNX members in various lower vertebrates and showed that the stem species of chordates most likely harbored a single gene copy, and that the entire Runt locus was triplicated in the course of evolution of chordates. Runx expression was examined during amphioxus development and was localized in ancient skeletal elements such as the notochord.

Importantly, RUNX proteins are often co-expressed with Hedgehog (Hh), another family of gene regulators essential for skeletogenesis and regulation of processes such as limb outgrowth, chondrocyte differentiation, and endochondral ossification (Chung et al. 2001, Day and Yang 2008). In amphioxus, RUNX protein can directly bind and activate the Hh promoter (Yoshida et al. 2004). Moreover, in primitive chordate skeletons, Hh is co-expressed with RUNX in the tissues that are known to be essential during cartilage and bone formation: notochord, neural tube, and the gill gut (Meulemans et al. 2003, Rychel and Swalla 2007).

Another signaling circuit that is intimately involved in skeletal development is the SOX gene cluster, structured within the 6 groups of genes (Soullier et al. 1999). SOX9 plays a critical role by initiating chondrogenesis and preventing subsequent maturation (Yamashita et al. 2009). Interestingly, Sox9 dominates over Runx2 in the mesenchymal precursor cells that are destined for a chondrogenic lineage: it acts to directly repress its activity (Zhou et al. 2006).

Recent phylogenetic analyses involving a cross-section of vertebrate ancestors have suggested that the occurrence of large-scale genomic events such as duplications might have acted as a prerequisite for creating the main components of cartilage and mineralized bone (Dehal and Boore 2005). The contribution of such events to creating the complexity typical of vertebrates is still debated, but large genetic effects do have an ability to drive the complexity of particular core genetic families, such as Sox and Runx (Donoghue et al. 2006). Thus, the basic genetic cassette may already have existed in protochordates—the ancestors of vertebrates—and then successively changed through gene duplications, domain shuffling, and other changes in the genome. This might have led to the formation of aggrecan, osteonectin, secretory calcium-binding phosphoproteins (SCPPs), and genes involved in Ca binding (Kawasaki and Weiss 2003, Wada 2010).

Recently, an evolutionary scenario was proposed for the assembly of the molecular machinery involving RUNX2 and the vitamin D3 receptor (VDR) in vertebrates (Marchellini et al. 2010). VDR is one of the nuclear hormone receptors, an evolutionarily related family of proteins that mediate the interaction between ligands and gene expression in all animals (Escriva et al. 2004, Bertrand et al. 2004). Vitamin D3 enables calcium and phosphate absorption from the gut, and its deficiency results in impaired bone mineralization and leads to bone diseases such as rickets and osteomalacia (Jurutka et al. 2007). In the lineage leading to amniotes, the widespread distribution of VDR probably contributed to co-expression with RUNX members and interaction with them in a variety of tissues, particularly in osteoblasts (Marchellini et al. 2010). In a series of biochemical experiments, VDR was found to interact directly with 3 Runx variants in the nuclei of rat cells stimulated with vitamin D3, demonstrating that the interaction between VDR and RUNX2 has played an important role in the mammalian lineage. Following their co-expression in the developing skeleton, the interaction between RUNX2 and VDR became involved in further definition of the features of the mammalian skeleton (Shen and Christakos 2005, Lian et al. 2006).

Finally, several other molecular pathways have played a role in shaping the vertebral skeleton and are indispensable for its formation and functioning, such as BMP, Wnt, Notch, FGF, and numerous others, with receptors that signal from the plasma membrane and that are regulated by networks of extracellular and intercellular factors (Ben-Shlomo et al. 2003, Wan and Cox 2005, Blitz and Cho 2009, Erwin and Davidson 2009).
